# Impact of preoperative laboratory frailty index on mortality and clinical outcomes in older surgical patients with cancer

**DOI:** 10.1038/s41598-022-13426-4

**Published:** 2022-06-02

**Authors:** Yoonjoo Kim, Kijun Song, Chang Moo Kang, Hyangkyu Lee

**Affiliations:** 1 Department of Nursing, Graduate School, Yonsei University, Seoul, South Korea; 2 Department of Nursing, College of Healthcare Sciences, Far East University, Eumseong-gun, Chungcheongbuk-do South Korea; 3 Mo-Im Kim Nursing Research Institute, College of Nursing, Yonsei University, Seoul, South Korea; 4 Division of HBP Surgery, Department of Surgery, Yonsei University College of Medicine, Seoul, South Korea; 5 Pancreatobiliary Cancer Center, Yonsei Cancer Center, Severance Hospital, Seoul, South Korea

**Keywords:** Biomarkers, Oncology, Risk factors

## Abstract

Frailty in older patients is associated with poor postoperative outcomes. The use of uncomplicated frailty measurement tools is preferred in busy clinical settings. Therefore, we validated the frailty index using routine laboratory data and the surgical outcomes of older patients with cancer who underwent cancer resection. We retrospectively analyzed 9015 patients aged 65 years and older who underwent cancer resection at a single tertiary hospital. Based on electronic-medical-record data regarding preoperative blood test results and vital signs, Laboratory Frailty Index (FI-Lab) scores were generated to measure preoperative frailty. The associations of FI-Lab with postoperative length of stay (LOS), readmission within 30 days, intensive care unit (ICU) admission within 30 days, and mortality were evaluated. The mean FI-Lab score of the 9015 patients was 0.20 ± 0.10. Increased FI-Lab scores (0.25–0.4; > 0.4) were associated with longer LOS, increased readmission within 30 days of surgery, ICU admission, and increased mortality, compared with FI-Lab scores < 0.25. The FI-Lab score, as a frailty indicator, was able to predict the risk of poor postoperative outcomes. Therefore, the FI-Lab is a potentially useful tool for assessing preoperative frailty in older patients with cancer in acute clinical setting.

## Introduction

Surgery is considered the optimal treatment for solid tumors; however, it can increase the postoperative risk of morbidity and mortality in older patients with cancer^1^. Therefore, factors other than age should be considered when predicting postoperative recovery in older patients with cancer^1,2^. Frailty is a clinical syndrome defined as condition that is vulnerable to maintaining homeostasis to stressors due to reduced physiological capacity^3–5^. Preoperative frailty in older patients with cancer has been found to be associated with poor surgical outcomes, such as greater length of hospital stay, complications, and mortality^6–9^. Several tools have been developed to measure frailty; nonetheless, there is no consensus regarding which tools are most useful in surgical patients. Among them, the frailty phenotype method^2^, and frailty index^10–13^ are widely used. However, both evaluation methods require skilled medical personnel; further, due to the long evaluation time and limited resources, it is difficult to apply them preoperatively in clinical practice to older patients with cancer^1,14^.

In recent years, to identify early frailty states that can increase the risk of clinically detectable frailty, a new indicator, the Laboratory Frailty Index (FI-Lab) score, has emerged and it is based solely on biomarkers of vital signs and the results of general blood tests, such as albumin, hemoglobin, cholesterol, sodium, and potassium^15–20^. This tool is well established in a sample of community residents^17–19^ and has been widely used to measure biological age in both western and eastern countries^15,16,21^. Studies have reported that, the FI-Lab demonstrated favorable consistency with the clinical frailty index^16,20^ in predicting mortality, number of hospitalization days, and frequency of hospital visits^15–18,20^, and the risk of mortality has reportedly increased with increasing FI-Lab scores in asymptomatic patients without clinically expressed frailty^17,18,22^. In addition, the FI-Lab score contributes to further quantification of risk beyond clinical evaluation for frailty^15,17,20,23^. Given these points, FI-Lab may potentially be a more objective measure than the frailty tools, which relies on subjective self-reported data. In acute care settings where various laboratory investigations are routinely performed and readily available in electronic health records, the FI-Lab captures frailty in a standardized way. Thus, FI-Lab can be easily applied to patients who are in the acute phase of surgery so that plans can be made to prevent complications and promote rapid recovery. Although many studies to date have reported frailty in cancer patients as a potential predictor^24,25^, few studies have measured frailty with the FI-Lab.

The aim of this study was to investigate the association between preoperative frailty and postoperative clinical outcomes and mortality in elderly patients with cancer. The FI-Lab scores were generated based on electronic-medical-record data. The following research questions were posed:

1. In elderly patients with cancer, can preoperative FI-Lab measure clinically indistinguishable frailty?
2. How is frailty in cancer patients identified by the FI-Lab related to postoperative length of stay (LOS), readmission within 30 days, intensive care unit (ICU) admission within 30 days, and mortality?

## Methods

This study was approved by the Institutional Review Board of Yonsei University Health System (Y-2020-0178). All methods were performed in accordance with the relevant guidelines and regulations, and informed consent was not required because anonymized data was used.

### Study design and participants

**Figure 1:**
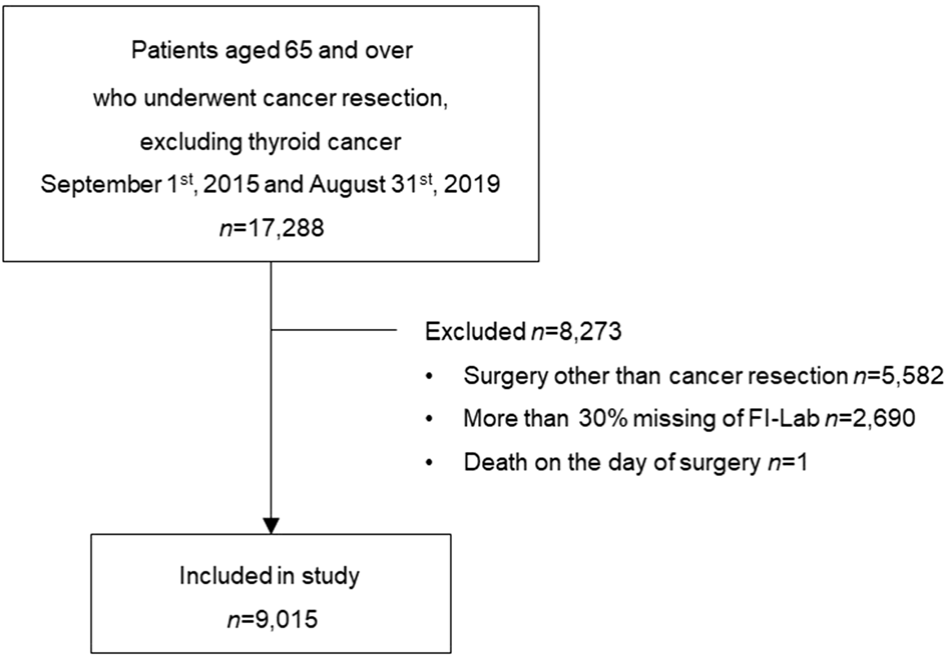
Selection of study patients. *FI-Lab* Frailty Index-Laboratory.

Between September 2015 and August 2019, this retrospective cohort study analyzed the electronic medical records of patients aged 65 years or older who underwent cancer resection at the Severance Hospital in South Korea. Although the highest incidence of thyroid cancer in Korea, people diagnosed with thyroid cancer were not included in this study because the 5-year relative survival rate was 100%^26^. Excluding those diagnosed with thyroid cancer, 17,288 patients aged 65 and older were confirmed to have undergone surgery. A total of 5582 patients who did not undergo cancer resection (examination or procedure) were excluded, and the FI-Lab scores of 2690 patients could not be calculated due to missing data. One patient who died on the day of surgery was excluded. The study’s final sample size was 9015 (Fig. 1).

### Laboratory frailty index

Frailty was assessed using FI-Lab scores^15^ generated based on previous reports from the Canadian Study of Health (CSHA)^18^ and the European Male Aging Study^17^. Abnormal levels of physiological parameters such as vital signs, routine blood and urine tests including creatinine, potassium, blood sugar, cholesterol, etc., are associated with health conditions and aging-related dysregulation of multiple organ systems (e.g., renal, liver, thyroid, hematological, inflammatory, coagulation, electrolyte)^27,28^. We identified 32 deficits from common blood test results and vital signs. Preoperative blood test results and vital-sign records measured initially on the day of surgery were used. Each deficit was coded using the normal reference range^15^ for each of the 32 variables (Appendix 1). If it was outside the normal range, it was assigned a score of “1”; if otherwise, it was assigned a scored as “0.” The FI-Lab score was obtained by summing the variable-deficit scores and dividing the total by the number of variables. For example, a person with a deficit in ten variables and no deficits in the other 21 variables of the 31-item FI-Lab tool would have an FI-lab score of 0.32 (10 divided by 31). FI-Lab scores range from 0 to 1; a higher score indicates greater frailty^15,18^. Frailty scores were categorized as follows: < 0.25, 0.25–0.4, and > 0.4^15,19^. FI-Lab scores were only calculated for subjects (at least 23 items out of a total of 32 items had to be measured) for whom < 30% of the variables were missing^16,18^.

### Postoperative health outcomes

Surgical outcomes included the following: postoperative LOS, readmission and ICU admission within 30 days after surgery, and overall survival. Based on a database recording secondary postoperative events that occurred within the first 30 days after surgery for surgical quality improvement^29^, readmissions and ICU admissions were defined within 30 days after surgery this study.

### Statistical analysis

The demographic and clinical characteristics of subjects are represented as means, standard deviations (SDs), frequencies and percentages using descriptive statistics. The comparison across groups were used to verify normality with QQ-plot, followed by analysis of variance and chi-square tests. Adjustments were made for age at surgery, sex, number of comorbidities, operating room duration, and cancer stage based on existing literature and clinical importance^15,30^. Postoperative LOS was defined as the number of days from the operation day to the discharge date, and linear regression analysis was used. Logistic regression analysis was performed in cases of unplanned readmission or ICU admission within 30 days of surgery considered to have an event. After checking assumption of proportionality, the effect of FI-Lab scores on mortality was investigated using Cox’s regression model. The Kaplan–Meier survival curve was plotted by FI-Lab group from the day of surgery to the day of death, and statistical significance was evaluated using the log-rank test. Statistical significance was offset at a two-tailed *p* value < 0.05. All analyses were conducted using SPSS software (version 26.0; IBM Corp., Armonk, NY, USA).

## Results

A total of 9015 subjects were included in the analysis. The mean age at the time of surgery was 72.3 years (standard deviation [SD] 5.3), and 65.5% of the patients were men. The FI-Lab mean was 0.20 (SD 0.10). The observed FI-Lab score range was 0.00–0.71; 6291 (69.8%), 2364 (26.2%), and 360 (4%) patients had scores within the “< 0.25”, “0.25 – 0.4”, and “> 0.4” categories, respectively. Of all patients, 47.8% had gastrointestinal cancer, and 17.4% had malignant neoplasms of the genital organs. The higher the FI-Lab score, the greater the frequency of patients with malignant neoplasms of gastrointestinal and genital organs (Table 1).

**Table 1:** Demographic and clinical characteristics of 9015 patients.

Characteristic	Preoperative FI-Lab; no. (%) of patients			*p* value
	< 0.25 *n* = 6291	0.25–0.4 *n* = 2364	> 0.4 *n* = 360	
FI-Lab, mean ± SD	0.15 ± 0.05	0.30 ± 0.04	0.47 ± 0.05	< 0.001
Age at surgery, years, mean ± SD	71.9 ± 5.1	73.2 ± 5.7	74.6 ± 6.2	< 0.001
Sex, male	4,195 (66.7)	1,471 (62.2)	242 (67.2)	< 0.001
**Number of comorbidity**				< 0.001
0	4321 (68.7)	1332 (56.3)	155 (43.1)	
1	1401 (22.3)	602 (25.5)	103 (28.6)	
2	447 (7.1)	298 (12.6)	53 (14.7)	
≥ 3	122 (1.9)	132 (5.6)	49 (13.6)	
**Cancer type**				0.050
Gastrointestinal	2878 (45.8)	1200 (50.8)	232 (64.4)	
Genital organ	1256 (20.0)	294 (12.4)	15 (4.2)	
Urinary tract	768 (12.2)	393 (16.6)	61 (17.0)	
Lung and bronchus	661 (10.5)	214 (9.1)	15 (4.2)	
Breast	461 (7.3)	145 (6.1)	5 (1.4)	
Other	266 (4.2)	118 (5.0)	32 (8.8)	
Operating room duration, min, mean ± SD	168.8 ± 113.0	191.1 ± 138.9	228.5 ± 170.0	< 0.001
**Cancer stage**				< 0.001
Stage 0/1	2,391 (39.0)	709 (30.0)	62 (17.2)	
Stage 2	1417 (22.5)	524 (22.2)	86 (23.9)	
Stage 3	1138 (18.1)	459 (19.4)	60 (16.7)	
Stage 4	312 (5.0)	226 (9.6)	55 (15.3)	
Unstaged/unknown	1,033 (16.4)	446 (18.8)	97 (26.9)	
**ECOG PS, *n* = 3665**	*n* = 2598	*n* = 946	*n* = 121	< 0.001
0	2494 (96.0)	872 (92.2)	104 (86.0)	
1	75 (2.9)	57 (6.0)	12 (9.9)	
2	21 (0.8)	13 (1.4)	3 (2.5)	
3	1 (0.0)	3 (0.3)	2 (1.6)	
4	7 (0.3)	1 (0.1)	0	
Postoperative LOS, day, mean ± SD	6.96 ± 8.6	9.6 ± 11.7	19.6 ± 26.5	< 0.001

*ECOG PS* eastern cooperative oncology group performance status, *FI-Lab* Frailty Index-Laboratory, *LOS* length of stay, *SD* standard deviation. Ohers indicated Head and neck cancer, hematologic malignancy, melanoma and bone cancer.

The mean (SD) postoperative LOS was 8.2 (11.1) days. Higher FI-Lab scores (FI-Lab score of 0.25–0.4: adjusted β 1.41, 95% confidence interval [CI] 0.95–1.88; FI-Lab scores > 0.4: adjusted β 9.45, 95% CI 8.40–10.50) were associated with longer LOS than FI-Lab scores < 0.25 after adjusting for age at surgery, sex, number of comorbidities, operating room duration, and cancer stage (Table 2).

**Table 2:** Association between preoperative frailty and surgical outcomes.

Outcome	Preoperative FI-Lab		
	< 0.25 *n* = 6291	0.25–0.4 *n* = 2364	> 0.4 *n* = 360
**Postoperative length of stay**			
Adjusted β (95% CI)	– (ref)	1.41 (0.95–1.88)	9.45 (8.40–10.50)
**Readmission within 30 days of surgery**			
No. (%) of patients	795 (12.6)	364 (15.4)	70 (19.4)
Adjusted OR (95% CI)	1.00 (ref)	1.20 (1.04–1.38)	1.49 (1.12–1.98)
**ICU admission within 30 days of surgery**			
No. (%) of patients	578 (9.2)	448 (19.0)	144 (40.0)
Adjusted OR (95% CI)	1.00 (ref)	1.70 (1.47–1.97)	3.58 (2.77–4.63)
**Mortality**			
No. (%) of patients	348 (5.5)	369 (15.6)	108 (30.0)
Survival time, month, mean (95% CI)	58.05 (57.76–58.34)	54.91 (54.24–55.58)	44.59 (42.11–47.08)
Adjusted HR (95% CI)	1.00 (ref)	1.75 (1.49–2.06)	4.29 (3.41–5.40)

All regression models adjusted for age at surgery, sex, number of comorbidity, operating room duration, and cancer stage. β coefficient after linear regression, OR after logistic regression, mean after Kaplan–Meier method, HR after Cox regression.

*CI* confidence interval, *FI-Lab* Frailty Index-Laboratory, *HR* hazard ratio, *ICU* intensive care unit, *OR* odds ratio; *Ref* reference.

Within 30 days of surgery, 1229 patients (13.6%) were unplanned readmitted. After adjusting for age at surgery, sex, number of comorbidities, operating room duration, and cancer stage, FI-Lab scores of 0.25–0.4 and > 0.4 were associated with readmission after surgery within 30 days (adjusted odds ratio [OR] 1.20, 95% CI 1.04–1.38, and 1.49, 95% CI 1.12–1.98, respectively) compared those > 0.25 (Table 2).

Within 30 days of surgery, 1,170 patients (13.0%) were admitted to the ICU, and the mean (SD) LOS was 2.2 (4.9) days. This outcome was observed in 40.0% (*n* = 144) and 19.0% (*n* = 448) of patients with frailty scores in the > 0.40 and 0.25–0.40 categories, respectively; however, it was observed in only 9.2% (*n* = 578) of patients with frailty scores < 0.25 (*p* < 0.001; Table 2). In the adjusted analysis, FI-Lab scores of 0.25–0.4 (adjusted OR 1.70, 95% CI 1.47–1.97) and > 0.4 (adjusted OR 3.58, 95% CI 2.77–4.63) were associated with postoperative ICU admission within 30 days (Table 2).

**Figure 2:**
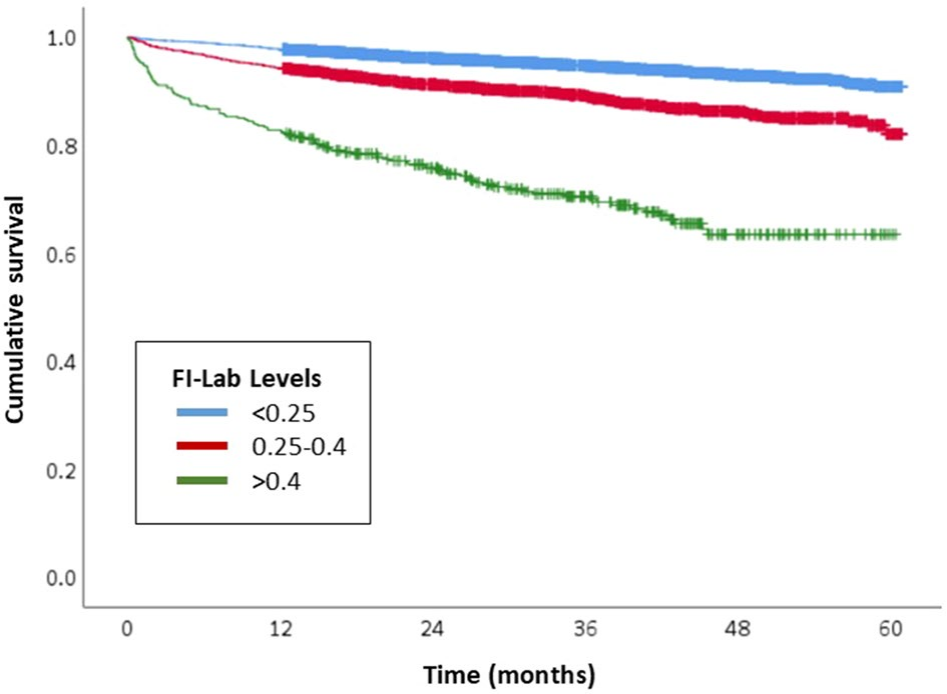
Kaplan–Meier survival curves among older surgical patients with cancer separated by frailty group.

During the follow-up period, 8.0% (*n* = 725) of the participants died. The mean (range) postoperative follow-up period for survivors was 34.7 (0.1–60.9) months. After adjusting for age at surgery, sex, number of comorbidities, operating room duration, and cancer stage, FI-Lab scores of 0.25–0.4 and > 0.4 were associated with an increased risk of mortality about twice (adjusted hazard ratio [HR] 1.75, 95% CI 1.49–2.06) and four times (adjusted HR 4.29, 95% CI 3.41–5.40), respectively, compared to FI-Lab scores < 0.25 (Table 2; Fig. 2).

## Discussion

This study had that patients’ frailty status may serve as an important preoperative indicator of postoperative patient outcomes in older patients undergoing cancer resection. FI-Lab scores, based on 32 routine blood tests and vital signs, were used to assess preoperative frailty in 9015 older patients who underwent cancer resection. A higher FI-Lab score was found to be associated with increased postoperative LOS, unplanned readmission and admission to ICU within 30 days, and mortality risk. Consequently, the FI-Lab score can be regarded as an effective tool as a prognostic factor in elderly patients considering cancer surgery. It also highlights the need to develop and evaluate strategies to improve outcomes by stratifying preoperative frailty to improve outcomes in cancer surgery patients in the presence of frailty.

A few previous studies conducted in surgical settings, it is widely accepted that frailty is a risk factor or unplanned readmission, postoperative complications, and high mortality^31–33^; however, most of them measured clinical frailty. For example, in a 2018 study of patients aged 65 years and older who underwent emergency abdominal surgery, frail patients, as defined using the clinical frailty scale, were predicted to be at four and three times the risk of 30 days and 6 months of readmission or death, respectively^8^. In the oncologic setting, compared comprehensive geriatric assessment (CGA) and FI, which are widely used for preoperative clinical frailty evaluation in patients with colon cancer. CGA predicted short-term surgical risk and survival, and FI as compared to CGA showed prognostic accuracy in predicting surgical 1-year mortality^34^. In addition, the modified frailty index derived from the cumulative deficit model was associated with increased major complications and readmissions within 30 days after surgery in colorectal cancer patients^35^. Few studies have evaluated cancer patient outcomes using the FI-Lab score. In a study of 306 patients aged 65 years and older who were hospitalized in elderly wards, both FI-Lab and clinical frailty scores (FI-CGA) were used^20^. A clear relationship between the FI-Lab score and mortality was reported, and FI-Lab demonstrated the main characteristics of clinical frailty. In addition, older patients hospitalized in general internal medicine wards found that higher FI-Lab scores were associated with increased hospitalization days, readmission rates, and mortality risk^16^. Our findings corroborate and expand existing evidence regarding FI-Lab yielded from previous studies conducted in acute care settings.

Some studies have reported that higher FI-Lab scores increase the risk of mortality, even in people with few clinical detectable deficits^17,18,22^. FI-Lab scores based on biochemical/physiological markers are believed to represent the burden of preclinical or subclinical deficits^17^. Eastern Cooperative Oncology Group (ECOG) performance status is a widely used method to evaluate the functional status of cancer patients as a major prognostic factor^36,37^. Performance status selects and stratifies patients for inclusion in treatment trials, and evaluates the quality of survival and prognosis of cancer patients^37^. In our study, 86% of patients in the group with the highest frailty exhibited grade 0 ECOG Performance Status of fully active persons (able to carry on all pre-disease performance without restriction)^37^. In a sense, asymptomatic dysregulation measured using FI-Lab, provides intermediate link within cellular level damage, indicating that it can eventually extending to clinically detectable deficits^23,38^. Therefore, the FI-Lab score is believed to have the potential to identify early-stage frailty before more advanced symptoms develop.

Laboratory testing is an objective measure that does not impose no extra strain on health care providers for data collection. Measured more often when a patient experiences a health condition (e.g., preoperative) that may attract more attention to treat and care^39^. Abnormalities in laboratory tests may reflect unhealthy conditions and dysfunction of organ systems that contribute to the risk of death. Therefore, preoperative stratification using the FI-Lab can help clinicians monitor patients' medical condition to identify high-risk patients to devise better treatment strategies. It can guide discussions among surgeons, anesthesiologists, patients, and their families to optimize perioperative management, as well as screening the effects of pre-habilitation to reduce vulnerability to surgery and increase patient recovery^40,41^. Quantifying frailty using FI-Lab may be a useful tool for evaluating frailty in older patients with cancer in various institutions and surgical services, given its ease of use, sensitivity to change and generalizability.

In addition, extensive evidence shows the importance of sex differences in frailty^42–44^. Female are known to have a higher degree of frailty than male at all ages^43^. Frail female have a higher risk of readmission and lower survival rate than frail male^43^. However, a recent study estimating sex-specific mortality using frailty index in a population of Korean adults, it was reported that although female had a higher frailty index than male, the survival probability was significantly lower in male than in female^44^. Perhaps this association suggests that characteristics of biological, behavioral, and social sex may affect biological health outcomes. However, sex differences with frailty in cancer patients have been relatively unexplored. Therefore, further studies are needed to investigate the association between FI-Lab and sex-specific surgical outcomes in elderly cancer patients, and to identify different cut off values for FI-Lab for gender categories^45^.

This study has certain limitations. First, since all cancer resections, except for thyroid cancer, were targeted, further studies are required to generalize the results to specific cancer. Second, this study analyzed the medical records of a single institution’s comprehensive cancer hospital. Considering surgical specialization, the number of patients, the treatment of older patients with cancer, and other resources that may not be present in other institutions, the association between frailty and surgical outcomes might have been underestimated. Further, in our multivariable model, neoadjuvant or adjuvant treatments (chemotherapy, radiotherapy, etc.) that potentially affect the mortality rate of patients undergoing cancer resection were not included. Future investigation is needed to evaluate the outcome after more homogeneous cancer resection based on the FI-Lab score and neoadjuvant or adjuvant treatment.

It is difficult to assess clinical frailty in acute care hospitals. Our results demonstrated the usefulness of the FI-Lab score, where preoperative frailty proved helpful in the early identification of surgical outcomes. Frailty in older patients, as evaluated by FI-Lab, demonstrated higher risks of hospitalization after cancer resection, ICU admission, unplanned readmission within 30 days, and death. This indicates the possibility of evaluating a surgical patient’s frailty more accurately and optimizing the feasibility of surgery. Moreover, our findings motivate further research in this area.

## Supplementary Information

Supplementary Information.

## Data Availability

The datasets generated during and analyzed during the current study are not publicly available due to medical records but are available from the corresponding author on reasonable request.
