# Supplementary material for: Impact of preoperative laboratory frailty index on mortality and clinical outcomes in older surgical patients with cancer
Source: Sci Rep. 2022 Jun 2;12:9200. doi: 10.1038/s41598-022-13426-4 (PMC9163125; doi:10.1038/s41598-022-13426-4)
Supplement: Supplementary file 1 — Supplementary Information. [file 41598_2022_13426_MOESM1_ESM.docx]

**Supplementary file: Laboratory frailty index (FI-LAB) items**

| Laboratory Measure | Low cut-off | High cut-off |
| --- | --- | --- |
| 1. Albumin (g/L) | 32 | 45 |
| 2. Alkaline phosphatase (U/L) | 20 | 130 |
| 3. Bicarbonate (mmol/L) | 21 | 28 |
| 4. Bilirubin, total (umol/L) | 2 | 21 |
| 5. Blood pressure- diastolic (mmHg) | 60 | 90 |
| 6. Blood pressure- systolic (mmHg) | 90 | 140 |
| 7. Blood urea nitrogen (mmol/L) | 2.9 | 8.2 |
| 8. C-reactive protein (mg/dL) | 0 | 1 |
| 9. Creatinine (umol/L) | Male: 60 | 110 |
|  | Female: 45 | 90 |
| 10. Direct HDL-Cholesterol (mmol/L) | 1.3+ | - |
| 11. Folate, RBC (nmol/L) | 376 | 1450 |
| 12. Glucose, serum (mmol/L) | 3.9 | 6.1 |
| 13. Glycohemoglobin levels (%) | 0 | 5.7 |
| 14. Hemoglobin (g/dL) | Male: 13.5 | 18 |
|  | Female: 12 | 16 |
| 15. Iron, refrigerated (umol/L) | 10.7 | 26.9 |
| 16. Lactate dehydrogenase LDH (U/L) | 100 | 190 |
| 17. Mean arterial pressure (mmHg) | 70 | 105 |
| 18. Mean cell volume (fL) | 80 | 96 |
| 19. Phosphorus (mmol/L) | 0.74 | 1.52 |
| 20. Platelet count SI (1000 cells/uL) | 150 | 450 |
| 21. Protein, total (g/L) | 60 | 78 |
| 22. Pulse (bpm) | 60 | 99 |
| 23. Pulse pressure (mmHg) | 30 | 65 |
| 24. Red cell distribution width (%) | 11.6 | 14.6 |
| 25. Segmented neutrophils percent (%) | 40 | 80 |
| 26. Sodium (mmol/L) | 136 | 142 |
| 27. Total calcium (mmol/L) | 2.3 | 2.74 |
| 28. Total Cholesterol (mmol/L) | 3.88 | 6.47 |
| 29. Triglyceride (mmol/L) | 0.11 | 2.74 |
| 30. Uric acid (umol/L) | Male: 240 | 510 |
|  | Female: 160 | 430 |
| 31. Vitamin B12, serum (pmol/L) | 118 | 701 |
| 32. Vitamin D (ng/mL) | 12 | 50 |
